# Venoplasty-facilitated cardiac implantable electronic device implantation and upgrade: feasibility and outcomes from a single center

**DOI:** 10.1007/s10840-026-02315-9

**Published:** 2026-03-31

**Authors:** Can Menemencioglu, Ugur Canpolat, Ahmet Hakan Ates, Can Sezer, Ahmet Kivrak, Kudret Aytemir

**Affiliations:** Department of Cardiology, Hacettepe University Faculty of Medicine, Sıhhiye, Ankara 06100 Turkey

**Keywords:** Cardiac implantable electronic devices, Venous occlusion, Venoplasty

## Abstract

**Background:**

Venous occlusion represents a significant challenge during de novo implantation or upgrading of cardiac implantable electronic devices (CIEDs), particularly in patients with chronic transvenous leads. Venoplasty may restore venous access while avoiding contralateral implantation or transvenous lead extraction (TLE).

**Objective:**

To evaluate the feasibility, safety, and procedural outcomes of venoplasty in patients with venous obstruction undergoing de novo CIED implantation or device upgrade.

**Methods:**

In this single-center retrospective study, 12 patients (median age 69 years; 75% male) who underwent venoplasty for total or subtotal venous occlusion identified by venography between December 2022 and December 2024 were included. Two patients underwent de novo CIED implantation and ten underwent device upgrade procedures. Venoplasty was performed using antegrade or retrograde approaches under fluoroscopic guidance, followed by standard CIED implantation.

**Results:**

Total venous occlusion was present in six patients and subtotal occlusion in six. The most common sites of obstruction were the brachiocephalic and subclavian veins. Antegrade venoplasty was successful in 11 patients, while retrograde femoral access was required in one case. Successful venous recanalization and CIED implantation were achieved in all patients (procedural success rate: 100%) without procedural complications. One patient developed subacute thrombosis related to anticoagulant non-compliance during follow-up.

**Conclusion:**

Venoplasty is a feasible, effective, and safe strategy for managing venous occlusion during de novo CIED implantation or device upgrade, reducing the need for contralateral access or TLE. This approach should be considered in appropriately selected patients at experienced centers.

**Supplementary Information:**

The online version contains supplementary material available at https://doi.org/10.1007/s10840-026-02315-9.

## Introduction

The number of cardiac implantable electronic device (CIED) implantations has steadily increased over recent years [1]. Venous occlusion represents a significant procedural challenge during CIED implantation, although it is rarely encountered during de novo procedures [2]. It occurs more frequently during device upgrade or revision procedures in patients with chronic transvenous leads. Lead-related venous occlusion (LRVO), defined as luminal narrowing exceeding 50% and occasionally complete vessel obstruction, remains a condition with incompletely understood mechanisms and risk factors [3, 4]. Although symptoms such as ipsilateral arm swelling, paresthesia, or pain may occur, LRVO is often asymptomatic due to the development of venous collateral circulation, even in cases of complete occlusion [5]. Consequently, asymptomatic LRVO is commonly diagnosed incidentally by venography during revision or upgrade procedures of existing CIED systems.

The conventional management strategy in patients with venous obstruction involves contralateral device implantation. However, this approach may lead to abandoned leads on the obstructed side and increase the risk of venous occlusion at the new implantation site, potentially resulting in bilateral venous obstruction. Transvenous lead extraction (TLE) followed by reimplantation through a new venous route represents an alternative strategy, but the presence of venous stenosis may increase procedural complexity and complication risk [6]. Venoplasty, a percutaneous balloon angioplasty technique that restores ipsilateral venous access, has emerged as a valuable alternative with potentially lower cost and fewer complications compared with other strategies [7]. In patients requiring de novo CIED implantation or device upgrade in the presence of unilateral or bilateral venous occlusion, venoplasty may provide a feasible and effective means of re-establishing venous access. However, data regarding the procedural feasibility and safety of venoplasty in real-world CIED upgrade and de novo implantation settings remain limited.

In the present study, we aimed to evaluate the feasibility, safety, and procedural outcomes of venoplasty in patients with venous obstruction identified during de novo CIED implantation or upgrading procedures.

## Methods

### Study population

Between December 2022 and December 2024, consecutive patients who underwent venoplasty for venous occlusion identified during de novo CIED implantation or device upgrade procedures at our center were retrospectively enrolled. Inclusion criteria consisted of patients requiring new lead placement in whom venous occlusion was confirmed by contrast venography. Among 12 included patients, venoplasty was performed during de novo CIED implantation in two cases, whereas the remaining ten patients underwent venoplasty during device upgrade procedures for pre-existing CIED systems. Baseline demographic characteristics and medical histories were obtained from electronic hospital records. The study was conducted in accordance with the Declaration of Helsinki and was approved by the Institutional Ethics Committee. Written informed consent was obtained from all patients prior to the procedures.

### Venoplasty and CIED implantation procedure

**Fig. 1:**
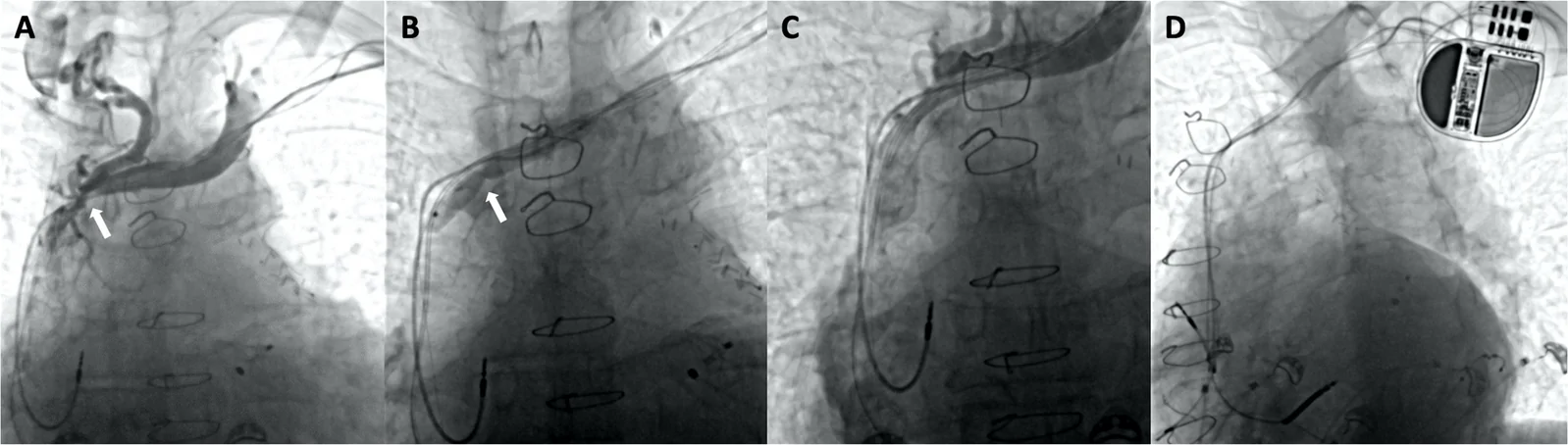
**A** Venography revealed a subtotal occlusion at the brachiocephalic vein-SVC junction **B** After crossing with 0.035 hydrophilic coated guidewire, the venoplasty was performed, upon observing a tight stenosis (white arrow), the balloon was inflated up to the rated burst pressure **C** Control venography following venoplasty revealed a significant increase in venous flow **D** Final fluoroscopic image showing that successful implantation of coronary sinus lead following restoration of venous patency

**Fig. 2:**
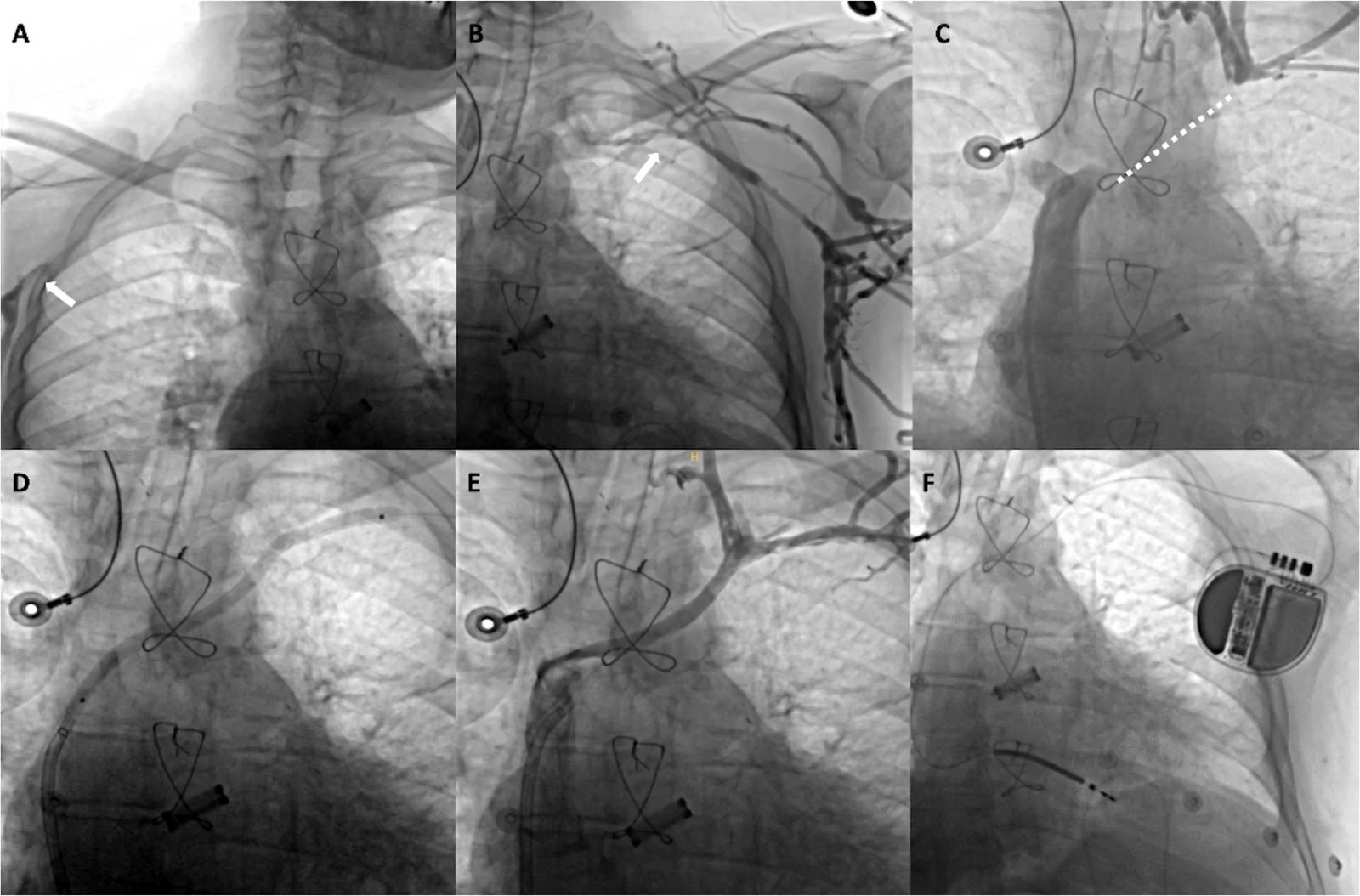
**A**,** B** Venography showed a total occlusion in the right axillary vein and the left subclavian vein. **C** After failing to cross the lesion via an antegrade approach, selective venography was performed through the long steerable sheath placed from the right femoral vein, which delineated the length of the venous occlusion. **D** After successfully crossing the subclavian lesion retrogradely, the balloon was inflated to the nominal pressure. **E**: Follow-up venography revealed restored flow. **F** Fluoroscopic image after VVI-ICD implantation

All procedures were carried out in a non-hybrid intervention room with cardiovascular surgery on standby. Initial non-selective venography was performed via the ipsilateral antecubital vein in all patients and additionally from the contralateral side in index cases using 20 mL of undiluted contrast medium to evaluate upper-extremity venous patency (Supplementary Video 1-left). Venous obstruction was classified as total occlusion in the absence of antegrade flow and/or presence of collateral circulation, and as subtotal occlusion when stenosis severity ranged between 75% and 99%. After sterile preparation, local anesthesia was administered using 20 mL prilocaine. Under intravenous deep sedation with midazolam and fentanyl, axillary vein access was obtained under fluoroscopic guidance using an 18-G needle. Following venous access confirmation, a 0.035-inch J-tip guidewire was advanced and a 6 F sheath was introduced. Selective venography was subsequently performed to delineate the occlusion characteristics (Supplementary Video 1-right). Crossing of the occluded segment was initially attempted using a 0.035-inch hydrophilic-coated guidewire (GlideWire, Terumo Interventional Systems, Somerset, NJ) employing a guidewire looping technique (Supplementary Video 2, Fig 1). In cases of unsuccessful crossing or complete occlusion, chronic total occlusion (CTO)-dedicated guidewires commonly used in coronary or peripheral interventions were utilized. When required, a coronary microcatheter or peripheral support catheter facilitated guidewire advancement. After successful lesion crossing, intraluminal positioning of the guidewire was confirmed fluoroscopically and by contrast injection through the microcatheter or support catheter (Supplementary Video 3), and the wire was advanced toward the inferior vena cava. The hydrophilic wire was then exchanged for a 0.035-inch stiff guidewire to provide adequate support. If antegrade axillary access failed, retrograde crossing was performed via the femoral vein using a long steerable sheath (Supplementary Videos 4–6, Fig 2). Following successful crossing, percutaneous transluminal angioplasty (PTA) was performed using appropriately sized balloons inflated at nominal pressure at the stenotic segment (Supplementary Video 7). If adequate luminal expansion was not achieved, balloon inflation was increased to rated burst pressure or optimized using a larger balloon (Supplementary Video 8). After each inflation, low-volume contrast venography was performed to assess residual stenosis and detect potential complications (Fig. 1).

After successful venoplasty, the device pocket was prepared and patients underwent either standard de novo CIED implantation or device upgrade according to clinical indication. Post-procedural chest radiography and echocardiography were performed in all patients. Patients were hospitalized overnight for observation. All patients received oral anticoagulation therapy for at least three months following venoplasty. Device clinic follow-up was scheduled at 6-month intervals. Physical rehabilitation was recommended for patients with lymphedema (Fig. 2).

Study outcomes included procedural effectiveness and safety of venoplasty and CIED implantation. Procedural success was defined as successful venous recanalization allowing intended CIED implantation without major procedural complications.

### Statistical analysis

Continuous variables with normal distribution are presented as mean ± standard deviation (SD), whereas skewed variables are expressed as median (interquartile range). Categorical variables are reported as numbers and percentages. Statistical analyses were performed using SPSS version 28.0 (IBM Corp., Armonk, NY, USA).

## Results

A total of 12 patients underwent venoplasty for venous occlusion detected during de novo CIED implantation (*n* = 2) or upgrade of pre-existing CIED systems (*n* = 10) (Table 1). Nine patients (75%) were male, and the median age was 69 years (IQR: 49–74). Device upgrade indications included cardiac resynchronization therapy (CRT) in seven patients: conventional biventricular pacing (BiVP-CRT) in three patients, left bundle branch area pacing (LBBaP)-CRT in one patient, and LBBaP-optimized CRT (LOT-CRT) in three patients. A new right ventricular (RV) lead was implanted in two patients due to IS-1 lead cable fracture and inappropriate implantable cardioverter-defibrillator (ICD) shocks. One patient with a prior dual-chamber pacemaker underwent upgrade to LBBaP because of pacing-induced cardiomyopathy.

**Table 1:** Patients’ demographics and clinical characteristics (*n* = 12)

Patient	Age	Sex	Comorbidities	Existing CIEDs		
				Months	Type	Number of leads
1	57	Male	Atrial Flutter, COPD, TLE, HFrEF	88	ICD	2
2	49	Male	AV Block, HFrEF (PiCM)	185	PM	2
3	69	Male	CAD, Diabetes, Hypertension, HFrEF	40	ICD	1
4	74	Female	AF, Hypertension, Diabetes, HFrEF	101	ICD	2
5	72	Male	CAD, AF, HFrEF	28	ICD	2
6	64	Female	Hypertension, HFrEF	61	CRT	3
7	71	Male	CAD, HFrEF	104	ICD	1
8	69	Male	AF, MVR, HFrEF	30	ICD	2
9	66	Male	Diabetes, HFrEF	48	ICD	1
10	70	Female	CAD, HFrEF	140	CRT	3
11	73	Male	AF, MVR, HFrEF	-	-	-
12	60	Male	CAD, AVR, HFrEF	-	-	-

*AF* Atrial Fibrillation, *AV* Atrio-ventricular, *AVR* Aortic valve replacement, *CAD* Coronary artery disease, *COPD* Chronic obstructive pulmonary disease, *CRT* Cardiac resynchronization therapy, *ICD* Implantable cardioverter defibrillator, *MVR* Mitral valve replacement, *PiCM* Pacing-induced cardiomyopathy, *TLE* Transvenous lead extraction

Among the two patients undergoing de novo CIED implantation, bilateral venous occlusion was identified on venography. LOT-CRT was successfully implanted in one patient, whereas the other received a single-chamber ICD (Table 2).

**Table 2:** Procedural characteristics of the study cohort (*n* = 12)

Patient	Venous Access	Occlusion site	Baloon size	Device implanted	Fluoroscopy Time (min)	Procedure Time (min)
1	R-Axillary	Subclavian	12 × 40 × 135 mm	CRT-D upgrade	38.2	158
2	L-Axillary	Subclavian, Brachiocephalic	4.0 × 40, 7.0 × 80, 8.0 × 40, 12.0 × 40 mm	DDD-PM (via LBBaP)	30.6	120
3	R-Axillary	Subclavian, Brachiocephalic	4.0 × 40 mm	LOT-CRT	16.9	110
4	L-Axillary	Brachiocephalic-SVC junction	6.0 × 60 mm	CRT-D (via LBBaP)	18.4	84
5	L-Axillary	Brachiocephalic-SVC junction	5.0 × 60, 8.0 × 60 mm	CRT-D upgrade	38.4	142
6	L-Axillary	SVC	4.0 × 13 mm	RV lead	18.8	115
7	L-Axillary	Subclavian	6.0 × 40 mm	LOT-CRT-D	22.2	124
8	L-Axillary	Subclavian, Brachiocephalic	3.0 × 20, 7.0 × 40 mm	LOT- CRT-D	46	150
9	L-Axillary	Subclavian	5.0 × 150, 6.0 × 150,7.0 × 150 mm	CRT-D upgrade	23.5	81
10	L-Axillary	Brachiocephalic	4.0 × 60 × 130, 6.0 × 80 × 130, 6.0 × 150 × 130 mm	RV Lead	28	118
11	L-Axillary	Brachiocephalic-SVC junction	8.0 × 40, 12 × 20 mm	LOT- CRT	20	89
12	L-Axillary, Femoral	Subclavian, Brachiocephalic	5.0 × 120 × 130, 7.0 × 80 × 130, 8.0 × 60 × 130 mm	VVI-ICD	48.5	180

*CRT* Cardiac resynchronization therapy, *ICD* Implantable cardioverter defibrillator, *L* Left, *LOT* Left bundle branch pacing optimized, *R* Right, *RV* Right ventricle, *SVC* Superior vena cava

Total venous occlusion was present in six patients and subtotal occlusion in six, with the brachiocephalic and subclavian veins representing the most frequently affected sites. Venoplasty followed by CRT-D upgrade was performed via the right side in two patients and via the left side in ten patients (Table 3).

**Table 3:** Post-procedural anticogulant therapy and follow-up

Patient	Anticoagulant therapy			Follow-up Duration
	Drug name	Dose	Duration	
1	Apixaban	2 × 5 mg	∗	20 months
2	Rivaroxaban	1 × 20 mg	6 months	24 months
3	Rivaroxaban	1 × 20 mg	3 months	24 months
4	Edoxaban	1 × 60 mg	∗	25 months
5	Rivaroxaban	1 × 20 mg	∗	36 months
6	Rivaroxaban	1 × 20 mg	3 months	44 months
7	Rivaroxaban	1 × 20 mg	3 months	14 months
8	Warfarin	∔	*	8 months
9	Apixaban	2 × 5 mg	6 months	7 months
10	Rivaroxaban	1 × 20 mg	3 months	22 months
11	Warfarin	∔	∗	12 months
12	Warfarin	∔	∗	12 months

∗In patients who were receiving anticoagulant therapy prior to the procedure due to additional comorbidities, continuation of anticoagulation was planned

∔The warfarin dose was adjusted according to the target INR

Antegrade venoplasty was successful in 11 patients (91.7%). In one patient, after failure of the antegrade approach, retrograde venoplasty via femoral access was successfully performed. Venoplasty was performed following transvenous lead extraction (TLE) in one patient with a prior contralateral TLE and complete superior vena cava occlusion. Procedural success rate was 100%, with venous access restoration enabling intended device implantation in all cases without procedural complications or damage to existing CIED leads.

Routine post-procedural imaging to assess venous patency was not performed and was reserved for symptomatic cases. The details of post-procedural oral anticoagulation therapy was represented in Table 3. One patient developed left arm swelling and subacute thrombosis of the left subclavian vein one week after the procedure, likely related to non-compliance with oral anticoagulation therapy. The patient was receiving warfarin, and the international normalized ratio (INR) was outside the therapeutic range. Following dose adjustment, therapeutic INR levels were achieved, and follow-up ultrasonography at three months demonstrated thrombus regression.

During follow-up, no clinical evidence of venous re-occlusion was observed at 6- and 12-month visits. Device interrogations demonstrated stable and normal sensing and pacing parameters in all patients at both follow-up time points.

## Discussion

Venous occlusion remains a significant technical challenge during both de novo cardiac implantable electronic device (CIED) implantation and device upgrade procedures, particularly in patients with chronic transvenous leads. In this single-center cohort, we demonstrated that venoplasty is a feasible, safe, and effective strategy for restoring venous access in patients with both total and subtotal occlusions, enabling successful device implantation without procedural complications.

### Venous occlusion in CIED patients

Venous obstruction may develop over time following CIED implantation and has been associated with multiple factors, including absence of anticoagulation therapy, number and diameter of implanted leads, and advanced patient age, although published data remain inconsistent and underlying mechanisms are incompletely understood [8–11]. Due to the development of collateral venous circulation, most patients remain asymptomatic, and venous occlusion is frequently detected incidentally during revision or upgrade procedures.

Contralateral implantation represents a conventional management strategy but may result in abandoned leads and increase the risk of bilateral venous obstruction. Transvenous lead extraction (TLE) is an alternative approach to restore venous access; however, procedure-related risks, technical complexity, and higher costs necessitate careful patient selection [12]. These limitations have encouraged the exploration of less invasive strategies.

Although previously described, venoplasty remains underutilized in CIED practice. When performed in experienced centers, it allows restoration of ipsilateral venous access without damaging existing leads or increasing peri-procedural morbidity or mortality. Appropriate candidates include patients requiring de novo implantation or device upgrade with guidewire-crossable venous stenosis or occlusion and favorable anatomical characteristics, such as short- or intermediate-segment lesions with preserved distal venous runoff toward the superior vena cava (SVC) or right atrium. Patients with non-crossable chronic occlusions, extensive fibrosis, absence of central venous outflow, severe calcification, active infection, or prohibitive procedural risk are generally unsuitable for this strategy [13].

In our cohort, venoplasty achieved procedural success in all patients without complications, supporting its safety and effectiveness when applied in appropriately selected cases. Preprocedural venography played a central role in defining occlusion characteristics and procedural planning. Although computed tomography or ultrasonography may assist evaluation, invasive venography remains the preferred modality in patients undergoing venoplasty [5].

### Advantages and limitations of venoplasty

Venoplasty offers several clinical advantages. First, preservation of ipsilateral venous access maintains future vascular options and avoids contralateral implantation [13, 14]. Second, it reduces the need for immediate TLE, thereby minimizing risks such as vascular injury, cardiac perforation, and procedure-related mortality [15]. Third, venoplasty enables continuation of standard implantation techniques without surgical exposure or hybrid procedures, potentially shortening procedural duration and hospitalization while reducing healthcare costs [13, 14].

However, venoplasty also has limitations. Crossing chronic total occlusions may be technically demanding and requires operator expertise and dedicated equipment [16]. Potential complications include vessel rupture, embolization, or lead damage, although these events are uncommon in experienced centers [13, 14, 16]. Long-term venous patency after venoplasty remains insufficiently studied, and re-occlusion may occur. Additionally, reliance on fluoroscopy and contrast administration may limit use in patients with renal dysfunction or contrast allergy. Therefore, careful patient selection and procedural planning remain essential.

### Anatomical distribution and procedural considerations

Lead-related venous obstruction is increasingly encountered as CIED implantation rates rise, with reported incidence ranging from 5% to 30% [5, 17]. Consistent with prior studies, occlusions in our cohort predominantly involved the brachiocephalic and subclavian veins, whereas isolated SVC occlusion was uncommon [18, 19].

Our standard strategy involves fluoroscopy-guided axillary vein puncture, which generally remains feasible when occlusion is proximal to the subclavian vein. Although distal venous access approaches have been described, they were not required in our cohort [7]. Antegrade venoplasty was successful in nearly all cases; retrograde femoral access was required in only one patient, highlighting the importance of alternative access strategies in complex occlusions.

Compared with TLE-mediated recanalization, venoplasty represents a less invasive alternative. Our institutional approach prioritizes venoplasty as the first-line strategy, reserving TLE-mediated recanalization for cases in which adequate venous flow cannot be restored. Notably, our center is a high-volume tertiary referral institution for TLE procedures (> 130/year), supporting the feasibility of this stepwise strategy.

### Special clinical scenarios

One patient with prior infection-related TLE and subsequent contralateral implantation presented with SVC occlusion requiring CRT-D upgrade. Successful restoration of SVC flow following venoplasty enabled device implantation, illustrating the potential applicability of this technique even in complex clinical scenarios.

Importantly, bilateral venous occlusion—an especially challenging situation—was identified in two patients undergoing de novo implantation, both of whom successfully received CIEDs following venoplasty. Data regarding venoplasty during de novo implantation remain limited (~ 13%) [20], representing a potentially novel contribution of the present study.

### Limitations

This study has several limitations, including the small sample size and retrospective single-center design. Additionally, long-term venous patency and re-occlusion rates were not systematically evaluated using routine imaging follow-up.

## Conclusion

Venous occlusion represents a major procedural challenge during de novo CIED implantation and device upgrade procedures. Venoplasty appears to be a feasible, effective, and safe technique for restoring venous access, potentially avoiding contralateral implantation or TLE-mediated recanalization. This strategy should be considered in appropriately selected patients treated at experienced centers with interventional expertise.

## Supplementary Information

Below is the link to the electronic supplementary material.

Supplementary Material 1. Video 1. Initial non-selective venography from the distal venous access showing the venous occlusion (left). However, selective venography from the axillary venous access demonstrated all the details of the venous occlusion segment and collateral circulation (right).

Supplementary Material 2. Video 2. When crossing the central cap of the occlusion was not possible, a coronary microcatheter or a peripheral support catheter was used to assist guidewire advancement. The venous occlusion is crossed using a 0.035`` hydrophilic-coated guidewire (GlideWire, Terumo Interventional Systems, Somerset, NJ) by the guidewire looping technique.

Supplementary Material 3. Video 3. After successful crossing of the venous occlusion, the intraluminal course of the guidewire was verified by administering contrast media from the coronary microcatheter or peripheral support catheter.

Supplementary Material 4. Video 4. Retrograde approach in a patient with failed antegrade crossing of the venous occlusion. Initial double contrast injection was showing the details of the venous occlusion and collateral circulation.

Supplementary Material 5. Video 5. The venous occlusion was crossed via a retrograde approach using a long steerable sheath and a peripheral support catheter.

Supplementary Material 6. Video 6. Using the tip-in technique, the retrograde wire was advanced into an antegrade microcatheter within the antegrade guide catheter, and we successfully performed the venoplasty.

Supplementary Material 7. Video 7. A percutaneous transluminal angioplasty (PTA) balloon of appropriate size(s) was inflated to achieve an adequate luminal expansion.

Supplementary Material 8. Video 8. Optimization of the venous expansion with a larger size balloon angioplasty and a final contrast venography was performed to confirm adequate venous flow.

Supplementary Material 9.

## Data Availability

Data available on request from the authors.

## Declarations

### Use of AI for writing assistance

This case report did not utilize artificial intelligence (AI)-assisted technologies.
