# Supplementary material for: Venoplasty-facilitated cardiac implantable electronic device implantation and upgrade: feasibility and outcomes from a single center
Source: J Interv Card Electrophysiol. 2026 Mar 31;69(8):2293–9. doi: 10.1007/s10840-026-02315-9 (PMC13739992; doi:10.1007/s10840-026-02315-9)
Supplement: Supplementary file 1 [file 10840_2026_2315_MOESM9_ESM.docx]

**Supplementary Video Legends**

**Video 1**. Initial non-selective venography from the distal venous access showing the venous occlusion (left). However, selective venography from the axillary venous access demonstrated all the details of the venous occlusion segment and collateral circulation (right).

**Video 2**. When crossing the central cap of the occlusion was not possible, a coronary microcatheter or a peripheral support catheter was used to assist guidewire advancement. The venous occlusion is crossed using a 0.035`` hydrophilic-coated guidewire (GlideWire, Terumo Interventional Systems, Somerset, NJ) by the guidewire looping technique.

**Video 3**. After successful crossing of the venous occlusion, the intraluminal course of the guidewire was verified by administering contrast media from the coronary microcatheter or peripheral support catheter.

**Video 4**. Retrograde approach in a patient with failed antegrade crossing of the venous occlusion. Initial double contrast injection was showing the details of the venous occlusion and collateral circulation.

**Video 5**. The venous occlusion was crossed via a retrograde approach using a long steerable sheath and a peripheral support catheter.

**Video 6**. Using the tip-in technique, the retrograde wire was advanced into an antegrade microcatheter within the antegrade guide catheter, and we successfully performed the venoplasty.

**Video 7**. A percutaneous transluminal angioplasty (PTA) balloon of appropriate size(s) was inflated to achieve an adequate luminal expansion.

**Video 8**. Optimization of the venous expansion with a larger size balloon angioplasty and a final contrast venography was performed to confirm adequate venous flow.
